# Head and Neck Cancer Treatment during COVID-19 Pandemic: A Central Experience in Rome. Emergency Management, Infection Prevention and Control

**DOI:** 10.3390/cancers13010033

**Published:** 2020-12-24

**Authors:** Andrea Cassoni, Resi Pucci, Nicolò Mangini, Maria Teresa Fadda, Andrea Battisti, Filippo Giovannetti, Valentina Terenzi, Marco Della Monaca, Paolo Priore, Ingrid Raponi, Valentino Valentini

**Affiliations:** 1 Department of Oral and Maxillofacial Sciences, Sapienza University of Rome, Via Caserta 6, 00161 Rome, Italy; andrea.cassoni@uniroma1.it (A.C.); nicolo.mangini@uniroma1.it (N.M.); mariateresa.fadda@uniroma1.it (M.T.F.); valentina.terenzi@uniroma1.it (V.T.); marco.dellamonaca@uniroma1.it (M.D.M.); ingrid.raponi@uniroma1.it (I.R.); valentino.valentini@uniroma1.it (V.V.); 2 Oncological and Reconstructive Maxillofacial Surgery Unit, Policlinico Umberto I of Rome, D.A.I. testa e collo. Viale del Policlinico 155, 00161 Rome, Italy; andrea-batti@libero.it (A.B.); filippo.giovannetti@uniroma1.it (F.G.); paolo.priore@uniroma1.it (P.P.)

**Keywords:** head and neck cancer, COVID-19 infection, pandemic, risk of contagion, oral-maxillofacial surgeons, infection prevention and control

## Abstract

**Simple Summary:**

The COVID-19 or SARS-CoV-2 pandemic has been spreading in our country since the beginning of 2020. Despite the emergency patients with head and neck cancer could not wait for treatment. Surgery in head and neck tumors, which consists of resection and reconstruction, must be timely, considering crucial further adjuvant treatments. Furthermore, the therapeutic results and morbidity depend on the size and progression of the disease. The management protocol included: telephone triage, nasopharyngeal swab tested for SARS-CoV-2 RNA before hospitalization, application of infection prevention and control (IPC) policies. This preliminary study reports our experience in managing oncological patients during the COVID-19 pandemic, evaluating the efficacy of the safety and containment measures implemented in our unit for the protection of the whole staff as well. In our opinion, even though restrictions have to be developed first and foremost for the patient’s safety, medical and paramedical staff need to be protected as well.

**Abstract:**

The COVID-19 pandemic has significantly affected the surgical units, especially those operating on the airways. This study evaluates the series of patients with tumors of the head and neck treated by our unit during Phase-1 of the pandemic and the efficacy of the preventive measures implemented for protecting both the patients and staff. A screening program was administered to all the patients who had to undergo surgery. None of the patients tested and operated during Phase 1, between 10 March and 18 May 2020, were positive for COVID-19. A significant portion of the patients was suffering from tumors in advanced stages (T3 and T4). Two patients developed respiratory symptoms during their stay at the unit, so they were put in precautionary isolation and tested, but resulted negative for COVID-19. All the surgical department staff followed the Italian Ministry of Health’s prevention protocol and underwent serological testing. IgG and IgM were negative in everyone, thus confirming that nobody had been exposed to the virus. This study highlights the commitment to efficiently treating patients suffering from tumors of the head and neck region and confirms the effectiveness of the safety measures used to protect our patients and staff’s health.

## 1. Introduction

The COVID-19 pandemic has been spreading in our country since the beginning of 2020, profoundly influencing our health care system, National Health Service, also known as SSN (Servizio Sanitario Nazionale), which offers universal access to health care [1,2]. As expected, a boost in the number of intensive care unit (ICU) spots for COVID-19 patients was necessary to cope with the emergency. As most of the ICU spots were needed to treat COVID patients and considering the high risk of contagion, the elective surgical activities had to be postponed [3,4]. Despite the emergency, however, our patients with cancer could not wait any longer for treatment. It has been necessary to reassess cancer treatment guidelines for head and neck oncological patients, keeping in mind the risks associated with COVID-19 infection. Additionally, it must be considered that COVID-19 risks involve many levels, including health care workers, nosocomial transmission, the use of immunosuppressive therapy, institutions, and, in some locations, social resource deficiencies in health care rationing. Due to all these factors, traditional head and neck oncology treatment needed to be reassessed, taking into account the specific risks of surgery during the COVID-19 pandemic [5]. The international literature recommends considering the risk of a COVID-19 infection when planning surgery on oncological patients [6]. The Government of Italy has put many safety measures in place to contain the spread of the virus. Since March 9, only essential services were available for the entire population, through the #iorestoacasa (#stayhome) decree. Phase 1 of the quarantine ended on 18 May with a progressive reopening of the businesses. So far, these measures have avoided a breakdown of the health care system in central-southern Italy. As far as health care and hospital services are concerned, only emergencies and oncological cases were authorized to undergo surgical procedures [7]. However, no guidelines were released for any single special unit, resulting in some confusion about patient management, procedure scheduling, and hospitalization agenda. Surgical procedures involving the nasal, oral, and endotracheal mucosal regions expose surgeons to a high risk of contagion due to the high quantity of aerosolized virus in these areas [8]. Therefore, in these extraordinary times, the research on how to safely and effectively operate deserves our attention [9]. During this emergency, the literature recommends nonsurgical treatment for oncological patients when surgical and conservative approaches are both first-line options. Until the emergency and the multilevel risks last, we are favoring nonsurgical treatment for most mucosal squamous-cell carcinomas, for which surgical and conservative approaches are both first-line choices. When surgery is to be preferred, an accurate multidisciplinary assessment of the surgical treatment’s multilevel risks is advised, with a discussion on viable alternative nonsurgical treatments and the patient’s involvement in the decision-making process. When the only option is surgery, accurate preoperative planning is recommended, with the implementation of COVID-19-specific perioperative protocols, to achieve maximum safety both in the surgical treatment and oncological care [5]. Surgery in head and neck tumors, which consists of resection and reconstruction, must be timely, considering crucial further adjuvant treatments [5,10]. Furthermore, the therapeutic results and morbidity depend on the size and progression of the disease. The tumor’s progression can be prevented with a timely intervention, to avoid worsening the prognosis, which could make complete resection impossible without treatment [5,10]. In our unit, we decided to screen the oncological patients who were to undergo surgery and to put preventive measures in place for doctors, nurses, health care workers, and patients to avoid any risk of infection.

This preliminary study reports our experience in managing oncological patients during the COVID-19 pandemic, evaluating the efficacy of the safety and containment measures implemented in our unit for the protection of the whole staff as well.

## 2. Materials and Methods

This single-institution, retrospective study includes all the patients diagnosed with head and neck cancer (HNC) admitted to the Reconstructive Oncology Unit. Criteria for inclusion were as follows: (1) patients surgically treated at the Oncological and Reconstructive Maxillofacial Surgery Unit, Policlinico Umberto I in Rome, Italy, (2) between 9 March and 9 June 2020, (3) with definitive histological diagnosis of head and neck cancer. All the surgeons, nurses, and health care workers (HCWs) of the Maxillofacial Surgery Department were evaluated and monitored. Demographic characteristics were recorded. The patients were classified following the NCCN guidelines (2019) or the AJCC staging system (8th edition). Descriptive statistics were calculated using SPSS Statistics. Treatment characteristics included surgical resection, type of reconstruction, and complications, if occurred. The screening program, composed of telephone triage and nasopharyngeal swab (NPS), was administered to all the patients before admission. SARS-CoV-2 RNA was tested by reverse transcription-quantitative polymerase chain reaction (RT-qPCR) performed on the nasopharyngeal swab (NPS). Currently, this is the gold standard for testing for COVID-19 [11]. According to the hospital’s guidelines, in the event of a positive swab, the patient was to be admitted to the COVID-19 unit of the Infectious Diseases Department, where he/she would be staying until complete recovery—i.e., three consecutive negative nasopharyngeal swabs. On the other hand, if negative for the infection, the patient was to be admitted to the unit within 48 h of the NPS and instructed to wear a mask, wash their hands, and respect social distancing prescriptions for the duration of the stay. The patient’s temperature would be checked every 8 h, and their clinical symptoms monitored constantly. All the medical staff were to adopt the safety and health measures and respect social distancing; the staff followed the prevention protocol of the Italian Ministry of Health and WHO’s Operational planning guidelines to support country preparedness and response as infection prevention and control (IPC) [1,12]. Following the suggestions provided by Zeng et al. [9], personal protective equipment can be classified according to the situation and the type of contact, as shown in Table 1. 

Before surgery, all patients were negative for NPS. Nonetheless, health workers should wear masks, protective clothing, and disposable gloves even when treating ordinary patients, and the protection protocols for treating suspected or confirmed COVID-19 patients should be more stringent, using all the protective devices indicated in Table 2. All the surgical procedures were performed according to the recommendations in the relevant literature, as Zou et al. recommend [13,14], to decrease the risk of spreading the infection. The patients who developed respiratory symptoms during hospitalization were tested with a new nasopharyngeal swab and underwent BAL (broncho-alveolar lavage), HRCT chest, and examination by an infectious disease specialist. In the event of suspected infection, the patient would be precautionarily put in isolation while waiting for diagnostic confirmation. If positive, the patient would be transferred to the dedicated COVID-19 unit. The medical staff, nurses, and health care workers (HCWs) were to adhere to the hospital’s rules of sanitary surveillance, introduced in the prime ministerial decree of 23 February 2020. Starting on 15 May 2020, the unit’s staff underwent serological screening: enzyme-linked immunosorbent assay (ELISA) was performed on whole blood for specific IgG and IgM antibodies [15], and NPS was tested for SARS-CoV-2 RNA.

## 3. Results

Sixty patients were included in this study, 34 males (56.7%) and 26 females (43.3%), with a median age of 71 (range 44–90). A total of 41 patients had primary tumors, and 19 were suffering from recurrences. In 14 cases, biopsies were performed. In total, 46 patients underwent complete tumor removal, and various reconstructive techniques were used: 10 free flaps, 9 locoregional flaps (pectoralis major flap, temporalis flap, supraclavicular artery island flap), 12 local flaps (buccal fat pad flap, buccinator myomucosal flap), 15 primary closures. A total of 54 cases were diagnosed as oral squamous cell carcinoma (OSCC): 15 staged as T4, 18 as T3, 12 as T2, 9 as T1. The characteristics of the series are summarized in Table 2. 

All the patients were hospitalized after the nasopharyngeal swab (NPS), followed by reverse transcription-quantitative polymerase chain reaction (RT-qPCR), tested negative for SARS-Cov-2. Two patients developed respiratory symptoms during their stay at the unit, after the surgical treatment, so they were put in precautionary isolation, using all the recommended personal protective equipment (PPE), and tested, but resulted negative for COVID-19. Nine doctors, 10 nurses, and three HCWs were enrolled in the study, for a total of 22 subjects. The median age was 46, with a range of 28–64 years, and the females represented 54.5% of the sample (the characteristics of the sample are summarized in Table 2). All the medical and paramedical staff on duty between 9 March and 9 June tested negative on the serological IgG and IgM test and NPS. The staff joined the study voluntarily.

## 4. Discussion

**Figure 1:**
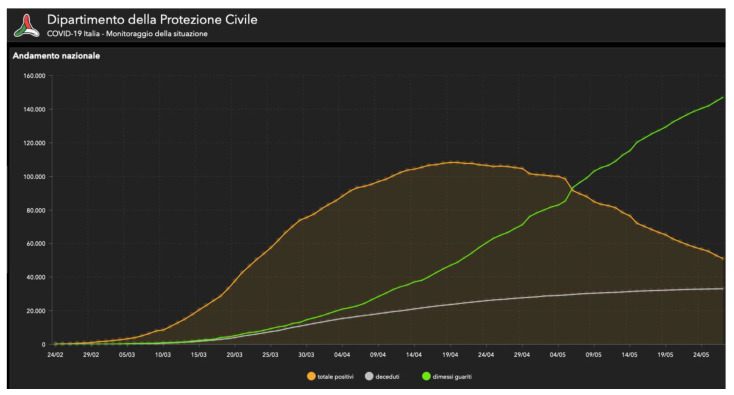
Graph describing the number of infections and deaths in Italy between February and May 2020. Yellow line: total positives; green line: healed and discharged; white line: dead. Official source: Ministero della Salute (Italian Ministry of Health). Last access: 28 May 2020. http://www.salute.gov.it/portale/nuovocoronavirus/dettaglioContenutiNuovoCoronavirus.jsp?lingua=italiano&id=5351&area=nuovoCoronavirus&menu=vuoto [17].

**Figure 2:**
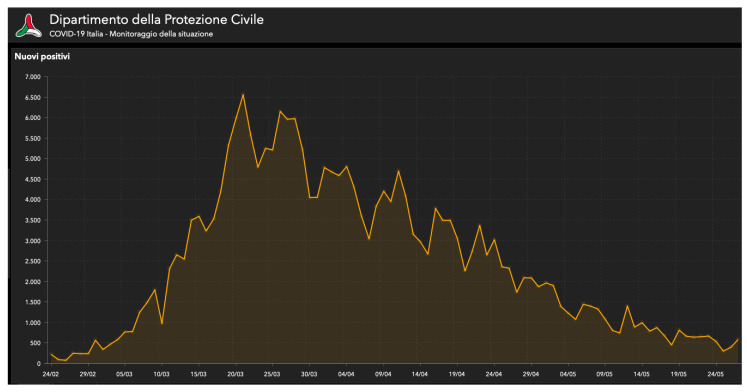
Graph showing the number of infections per day between February and May 2020 and highlighting the peak of infections between 20 and 30 March 2020. Official source: Ministero della Salute (Italian Ministry of Health). Last access: 28 May 2020. http://www.salute.gov.it/portale/nuovocoronavirus/dettaglioContenutiNuovoCoronavirus.jsp?lingua=italiano&id=5351&area=nuovoCoronavirus&menu=vuoto [17].

Infection with the novel coronavirus (SARS-CoV-2, which is the virus responsible for the coronavirus disease 2019, known as COVID-19) was first detected in Wuhan, China, in December 2019. The outbreak of COVID-19 remains ongoing and has been linked to more than 80,000 infected patients and more than 3000 deaths in China, as reported on 7 March 2020 [16]. The COVID-19 pandemic has been affecting our country during the last months, profoundly influencing our health system and economy [17]. The Italian government has put many safety measures in place in the hope of effectively containing the spread of the virus [2]. From 9 March to 18 May, only essential services were available for the entire population, and everybody promptly accepted the #iorestoacasa (#stayhome) decree. In Italy, 231,139 people were infected with COVID-19 and 33,072 died (Figure 1) between the beginning of the pandemic and 28 May [18]. The infection was at its peak between 20 and 30 March (Figure 2). Phase 2 began on 18 May, with a progressive reopening of the businesses. In Rome, 5614 cases were recorded until 28 May [18]. The COVID-19 pandemic has seriously affected safety in surgical practices, considering above all the risks of the specialists who operate on the airways, like oral-maxillofacial surgeons [8]. Surgical procedures involving the nasal, oral, and endotracheal mucosal regions expose surgeons to a high risk of contagion due to the high quantity of aerosolized virus in these areas [13]. Many patients cannot wait for the pandemic to be over to undergo surgery, such as those suffering from cancer [11,19]. In 2018, 354,864 new cases of oral and lip cancer were diagnosed worldwide, and 3967 new oral cancer cases in Italy, as reported by the International Agency for Research on Cancer (IARC) on the Global Cancer Observatory (GCO) platform. Every year, an estimated 177,384 people die of oral and lip cancer around the world, 1489 of whom in Italy [20]. In the case of head and neck tumors, the surgical treatment, consisting of resection and reconstruction, must be timely, to allow for any possible adjuvant treatment [21,22,23]. Moreover, therapeutic outcomes and morbidity hinge on the disease’s size and progression. A timely intervention prevents the tumor’s progression and avoids the worsening of the prognosis, which could make a complete surgical resection eventually impossible [21]. To hopefully increase our oncological patients’ functional and oncological outcomes [14,24], we need to operate on them within a few weeks since diagnosis. By analyzing the historical case series of our unit, we highlight our Hospital’s commitment to the management of COVID-19 patients. In our experience, the three fundamental elements for pandemic management were as follows: telephone triage, NPS tested for SARS-CoV-2 RNA before hospitalization, and application of infection prevention and control (IPC) policies. The IPC policies implemented in our Institution were based on respecting social distancing, hygiene standards—including frequent hand washing—and the use of PPE. PPE has been a crucial element to reduce the spread of the virus, but, especially in the first phase of the pandemic, there was a lack of these aids. Therefore, careful and correct use was essential. All elective surgeries were cancelled during the Phase 1 of the pandemic—also because of a massive reduction in the number of available spots in intensive care units—resulting in a lower number of patients. Although this study does not involve a large sample, the results suggest that the measures implemented to prevent and control the spread of the virus were effective. However, this is a preliminary study that we intend to extend to the next phases of the pandemic. In addition to the nasopharyngeal swab (NPS) followed by reverse transcription-quantitative polymerase chain reaction (RT-qPCR) performed on all patients before admission, all medical staff underwent serological testing, to test all those who came into contact with the patients included in the study [11]. In this way, by evaluating the medical staff’s immune status, we evaluated the adequacy of the protocol we had applied. Limiting human-to-human transmission—including secondary infections that can spread among health care workers—is one of the World Health Organization’s strategic priorities. In our opinion, even though restrictions have to be developed first and foremost for the patient’s safety, medical and paramedical staff need to be protected as well. 

## 5. Conclusions

The COVID-19 emergency requires continuous effort, and oral-maxillofacial surgeons cannot stop working, because they need to guarantee the best surgical treatments, despite the risks. The limitation of this preliminary study is the small size of the sample, but it has been useful in our clinical practice nonetheless. We have used effective measures to protect staff, reduced PPE wastage during the emergency phase, when it was in short supply, and adequately treated all patients. This is the picture of our activity in the worst months of the pandemic. In recent months, it has been essential to continue to treat patients with HNC, but, sometimes, many services, especially primary health care, have failed. The inability to schedule outpatient and screening visits will inevitably lead to many cases of diagnostic delay, and we will suffer the consequences of this pandemic also in our daily battle against cancer. 

## Figures and Tables

**Table 1:** Personal protective equipment (PPE) requirements for medical staff.

Usage	Surgical Mask	N95 Mask	Disposable Glovers	Isolation Gown	Protective Clothing	Disposable Shoe Covers
*Before surgery*	✓		✓			
*During surgery*						
Ordinary patients	✓		✓		✓	✓
Ordinary patients with tracheotomy	✓	✓	✓		✓	✓
*After surgery*						
Ordinary patients	✓		✓			
Ordinary patients with tracheotomy	✓	✓	✓		✓	✓
Confirmed/suspected patients	✓	✓	✓	✓	✓	✓

**Table 2:** Sample characteristics. SD: standard deviation, OSCC: oral squamous cell carcinoma, HCWs: health care workers. Simple descriptive statistics performed by SPSS Software.

Patient Population	Descriptive Statistics
*Sample size (n):*	60
*Sex:*	
Male	34 (56.7%)
Female	26 (43.3%)
*Age (median and range)*	71 (44–90)
*Type:*	
Primary	41 (68.3%)
Recurrence	19 (31.7%)
*Diagnosis of OSCC (n)*	54 (90%)
*Stage:*	
T1	9 (15%)
T2	12 (20%)
T3	18 (30%)
T4	15 (25%)
*Treatment:*	
Free flap	10 (16.7%)
Locoregional flap	9 (15%)
Local flap	12 (20%)
Primary closure	15 (25%)
Biopsy	14 (23.3%)
***Medical Staff***	***Descriptive Statistics***
*Sample size (n):*	22
Doctors	9 (41%)
Nurses	10 (45.4%)
HCWs	3 (13.6%)
*Sex:*	
Male	10 (45.5%)
Female	12 (54.5%)
*Age (median and range)*	46 (28–63)

## Data Availability

The data presented in this study are available on request from the corresponding author.
